# Supplementary material for: Cloud BioLinux: pre-configured and on-demand bioinformatics computing for the genomics community
Source: BMC Bioinformatics. 2012 Mar 19;13:42. doi: 10.1186/1471-2105-13-42 (PMC3372431; doi:10.1186/1471-2105-13-42)
Supplement: Additional file 1 — Supplementary 1 Cloud BioLinux software documentation in the form of a mini, self-contained website. Users need to download and uncompress the .zip file, and open through a web browser the "index.html" file available on the main directory. (ZIP 1823 kb). [file 1471-2105-13-42-S1.ZIP › Cloud-BioLinux-Package-Documentation/docs/cn3d.html]

Bio-Linux Software Documentation Pages

Back to search form

## cn3d

|  |  |
| --- | --- |
| Name | cn3d |
| Description | A three dimensional viewer for biological molecules. |
| Homepage | http://www.ncbi.nlm.nih.gov/Structure/CN3D/cn3d.shtml |
| Remote Documentation | http://www.ncbi.nlm.nih.gov/Structure/CN3D/cn3dtut.shtml   http://www.ncbi.nlm.nih.gov/Structure/CN3D/cn3dfaq.shtml |
